# Hidrox^®^ Roles in Neuroprotection: Biochemical Links between Traumatic Brain Injury and Alzheimer’s Disease

**DOI:** 10.3390/antiox10050818

**Published:** 2021-05-20

**Authors:** Marika Cordaro, Angela Trovato Salinaro, Rosalba Siracusa, Ramona D’Amico, Daniela Impellizzeri, Maria Scuto, Maria Laura Ontario, Roberto Crea, Salvatore Cuzzocrea, Rosanna Di Paola, Roberta Fusco, Vittorio Calabrese

**Affiliations:** 1 Department of Biomedical, Dental and Morphological and Functional Imaging, University of Messina, Via Consolare Valeria, 98125 Messina, Italy; cordarom@unime.it (M.C.); calabres@unict.it (V.C.); 2 Department of Biomedical and Biotechnological Sciences, University of Catania, 95124 Catania, Italy; trovato@unict.it (A.T.S.); mary-amir@hotmail.it (M.S.); marialaura.ontario@ontariosrl.it (M.L.O.); 3 Department of Chemical, Biological, Pharmaceutical and Environmental Sciences, University of Messina, 98166 Messina, Italy; rsiracusa@unime.it (R.S.); rdamico@unime.it (R.D.); dimpellizzeri@unime.it (D.I.); rfusco@unime.it (R.F.); 4 Oliphenol LLC., 26225 Eden Landing Road, Unit C, Hayward, CA 94545, USA; robertocrea48@gmail.com

**Keywords:** oxidative stress, inflammation, neurodegeneration

## Abstract

Traumatic brain injuries (TBI) are a serious public-health problem. Furthermore, subsequent TBI events can compromise TBI patients’ quality of life. TBI is linked to a number of long- and short-term complications such as cerebral atrophy and risk of developing dementia and Alzheimer’s Disease (AD). Following direct TBI damage, oxidative stress and the inflammatory response lead to tissue injury-associated neurodegenerative processes that are characteristic of TBI-induced secondary damage. Hidrox^®^ showed positive effects in preclinical models of toxic oxidative stress and neuroinflammation; thus, the aim of this study was to evaluate the effect of Hidrox^®^ administration on TBI-induced secondary injury and on the propagation of the AD-like neuropathology. Hidrox^®^ treatment reduced histological damage after controlled cortical impact. Form a molecular point of view, hydroxytyrosol is able to preserve the cellular redox balance and protein homeostasis by activating the Nrf2 pathway and increasing the expression of phase II detoxifying enzymes such as HO-1, SOD, Catalase, and GSH, thus counteracting the neurodegenerative damage. Additionally, Hidrox^®^ showed anti-inflammatory effects by reducing the activation of the NFkB pathway and related cytokines overexpression. From a behavioral point of view, Hidrox^®^ treatment ameliorated the cognitive dysfunction and memory impairment induced by TBI. Additionally, Hidrox^®^ was associated with a significant increased number of hippocampal neurons in the CA3 region, which were reduced post-TBI. In particular, Hidrox^®^ decreased AD-like phenotypic markers such as ß-amyloid accumulation and APP and p-Tau overexpression. These findings indicate that Hidrox^®^ could be a valuable treatment for TBI-induced secondary injury and AD-like pathological features.

## 1. Introduction

Traumatic brain injury (TBI) annually affects approximately 1.7 million people [1,2]. It is defined as mechanical injury and/or structural disruption of brain function resulting from rapid brain deceleration or acceleration or from striking the head with a hard object. Causes of TBI are motor vehicle accidents, falls, and assaults [2]. It is an heterogeneous disease with many different shades [3]. TBI can induce a host of emotional, physical, behavioral, and cognitive changes, and outcomes can range from total recovery to death or permanent disability [4]. It consists in a primary insult caused by biomechanical forces and a secondary injury that has a key role in tissue molecular damage.

TBI secondary injuries commonly include long-lasting neuropathologies such as neurodegenerative diseases and dementia [5,6]. Nowadays, the TBI-associated secondary insult is recognized as equally injurious as the primary damage because of the ensuring morbidity. Several reports describe impaired calcium influx, glutamate accumulation, elevated amyloid precursor protein (APP) expression, and neurotoxic inflammation [7,8]. These biochemical changes occur throughout the brain and induce neurodegeneration coupled with cognitive and motor impairments [9,10]. These secondary outcomes double the risk of developing Alzheimer’s disease (AD)-like symptoms [11]. AD is the most usual cause of dementia in the elderly, affecting 11% of those over the age of 65 years and around a third of those aged 85 and up [12]. Post-mortem brain tissues from AD patients showed multifocal axonal swelling, exacerbate inflammation, accumulated APP and amyloid β peptides [13]. Many in vivo studies show the development of AD pathology in the late stage of TBI, supporting the hypothesis that abnormal APP protein expression can be a hallmark of AD [14,15].

The TBI-induced pathological changes can shear axons, tear neurons, damage the vasculature, and disrupt neuronal circuits, leading to necrotic cell loss and apoptosis of surrounding cells [15].

The inflammatory cell response in the injured area involves the expression of many pro-inflammatory mediators and the overproduction of free radicals [3,16]. Several papers underline the role of oxidative stress in the damaged brain area [17]. Immediately after TBI, the superoxide anion is the most common detected free radical that supports the formation of other reactive nitrogen species/oxygen species (RNS/ROS) inducing lipid peroxidation [18]. Several lines of evidence display the important role of the nuclear factor–erythroid 2-related factor (Nrf2) in the injury induced by TBI. As a pleiotropic transcription factor, it protects cells from oxidative/cytotoxic damage, inducing detoxifying and antioxidant enzymes.

In unstressed conditions, the Kelch-like ECH-associated protein 1 (KEAP1) binds Nrf2 via its binding domain and sequesters it in the cytoplasm. In injured conditions, the increased ROS modify cysteines residues in KEAP1, thus modifying its conformation and reducing its affinity for Nrf2, which translocates into the nucleus. Once translocated, it combines with antioxidant response element (ARE) sequences, activates the Nrf2/ARE pathway, and then the gene expression of phase II detoxifying enzymes and antioxidases, such as heme oxygenase 1 (HO-1), SOD-1, and glutathione peroxidase 1 (GPx1), which protect cells from oxidative stress and a broad range of other toxins [19,20,21].

When the production of ROS exceeds the scavenging capacity of antioxidant response systems, extensive lipid peroxidation and protein oxidation occurs, causing oxidative damage, cellular degeneration, and even functional decline [22]. ROS also interact with nuclear factor κB (NFκB) that is known to be activated by the redox state of the cell in a number of pathologies [23,24,25]. Such activation can be inhibited by the use of antioxidants [26].

NFkB is tightly regulated. In physiological conditions, it is sequestered in the cytoplasm bound to the inhibitory protein IkB-α. Once IkB-α is degraded in response to oxidative and inflammatory stimuli, NFkB is free to translocate into the nucleus. NFkB activation increases the transcription of proinflammatory cytokines, and they in turn activate NFkB [27]. The positive feedback amplifies the inflammatory signals [28,29].

Thus, the development of anti-oxidative and anti-inflammatory strategies for the management of primary and secondary insults induced by TBI is a subject of great scientific interest.

Concerning this aspect, incoming studies report the positive effects of natural phytocomponents as bioactive molecules against neurodegenerative disease [30,31,32]. The Mediterranean Diet promotes a high intake of fruits and vegetables, leading to the reduction of saturated fats. In particular, one of the main components of the Mediterranean Diet is olive oil, and the main phytochemical contained in it is hydroxytyrosol. This molecule has been described as free-radical scavenger and an antioxidant with important antimicrobial, anticancer, anti-inflammatory, and neuroprotective activity [33,34,35]. Many in vivo studies show hydroxytyrosol beneficial effects in the brain: in a Huntington’s disease model, hydroxytyrosol protects the brain from oxidative damage by reducing lipid peroxidation and increasing glutathione (GSH) levels [36]; in a oligomeric acid Aβ_1-42_ + ibotenic acid-induced neural behavioral dysfunction model, hydroxytyrosol ameliorated the working and visuo-spatial memories, thus restoring signaling mechanisms in hippocampal neurons [37,38]. Recent data from our laboratory confirmed that Hidrox^®^, an aqueous extract of olive incorporating 40–50% of hydroxytyrosol, is a very effective anti-inflammatory agent and a powerful antioxidant.

In particular, Hidrox^®^ is a freeze–dried powder prepared from the aqueous portion of olives extracted from defatted olive pulps, a derivative obtained during the processing of *Olea europaea* L. for olive oil extraction [39]. A total of 12% of the Hidrox^®^ extract consists of polyphenols. Among these, the most abundant in HD is hydroxytyrosol, representing 40–50%, while oleuropein is present at 5–10%, tyrosol at 0.3%, and oleuropein aglycone and gallic acid at about 20% [40]. Hidrox^®^ counteracts the neurodegenerative processes characteristic of Parkinson’s disease [41]. Starting from this evidence, we evaluated the protective effects of Hidrox^®^ on TBI second damage and the propagation of the AD-like neuropathology.

## 2. Materials and Methods

### 2.1. Animals

Two-month-old Sprague-Dawley rats (Envigo, Milan, Italy) were used in this research. The University of Messina Review Board for animal care (OPBA) approved the study. All animal experiments agreed with the new Italian regulations (D.Lgs 2014/26), EU regulations (EU Directive 2010/63), and the ARRIVE guidelines.

### 2.2. Experimental Protocol

Animals were anesthetized with 1–2% isoflurane and maintained with a gas mask. TBI was performed as already described [15]. After scalp incision, craniectomy was performed, and coordinates of +0.2 mm lateral and −0.2 mm anterior to the midline were employed to impact the brain at the fronto-parietal cortex, reaching a depth of 1.0 mm below the dura matter layer at a velocity of 6.0 m/s. After the impact, the skin incisions were closed, and a 2% lidocaine jelly was applied to the lesion site. All animals were monitored post-operatively and kept hydrated.

### 2.3. Experimental Groups

Rats were randomized and assigned to the following groups (*n* = 20):

(1) Vehicle group: Rats were subjected to TBI as described above, and vehicle (saline) was administered by gavage for 4 weeks.
(2) Hidrox^®^ group: Rats were subjected to experimental TBI as described above, and Hidrox^®^ (10 mg/Kg) was administered 1 h after TBI and daily by gavage for 4 weeks.
(3) Sham group: Rats were subjected to the surgical procedures (anesthesia, craniectomy, and suturing), and vehicle (saline) was administered by gavage for 4 weeks.

The dose of Hidrox^®^ was based on previous experiments [41].

In order to evaluate the secondary injury induced by TBI, rats were sacrificed at 4 weeks after TBI induction.

Four weeks after TBI induction, behavioral tests were performed, and rats were sacrificed, collecting brain tissues for further analysis.

### 2.4. Morris Water Maze (MWM)

A circular blank water container (60 cm in height and 152 cm in diameter), filled with water (23 °C) to a profundity of 30 cm and with an escape platform of 10 cm of diameter was employed to perform the test. The platform, with the top 2 cm below the water surface, was placed in a quadrant of the tank and remained fixed during the experiment. Above the tank, a white curtain was drained around the pool, and four types of black paper with different forms were hung on the interior of the curtain. Each animal was subjected to a daily trial session for four days. A probe trial was performed 24 h after the last training session. The percentage of distance covered and the time spent in the target quadrant were recorded [42].

### 2.5. Elevated Plus Maze Test

The elevated plus maze apparatus consisted of two closed arms (50 × 10 × 40 cm) and two open arms (50 × 10 cm) connected by a central square (10 × 10 cm). The acquisition of memory was tested on the 27th day after TBI (test session). Animals were placed individually at one end of the open arm, facing towards the open end of the maze. The time of travel of the animal from the open arm to the closed arm was recorded as initial acquisition latency (IAL). The animal was allowed to explore the maze for 20 s after recording the IAL and then returned to the home cage. If the animal did not enter the enclosed arms within 90 s, it was pushed to one of the enclosed arms, and the IAL was recorded as 90 s. Retention of memory was assessed by placing the rat in an open arm, and the retention transfer latency (RTL) was noted on the 28th day after TBI (re-test session).

Based on the observed experience-dependent behavioral changes, the test and re-test results can suggest modulation of memory-related processes [43,44].

### 2.6. Determination of Reduced Glutathione Levels

The levels of reduced glutathione (GSH) were determined in brain tissues to evaluate the endogenous antioxidant defenses. Brain samples were homogenized with 0.2 M phosphate buffer (pH 7.6). Then, a trichloroacetic acid solution was added, and the mixture was centrifuged at 3900 g. 5,5′-dithiobis-(2-nitrobenzoic acid) was added, and samples were incubated at room temperature for 5 min. GSH levels were determined using a microplate reader at 412 nm [43].

### 2.7. Measurement of Superoxide Dismutase (SOD) Activity

Brain tissues were homogenized in Tris buffer (pH 8.2) and centrifuged at 13,000 rpm. TritonX-100 was added, samples were incubated at 4–8 °C for 20 min and then centrifuged at 10,000 rpm. Samples’ pyrogallol absorbance was measured for 10 min at 420 nm every 60 s [43].

### 2.8. Measurement of Catalase Activity

Brain tissues were homogenized in phosphate buffer at 1800 rpm, then hydrogen peroxide was added, and absorbance measured for 0–10 min at 240 min [43].

### 2.9. Measurement of Lipid Peroxidation

Brain tissues were homogenized in Hank’s balanced salt solution at 3000 rpm. Pellets were incubated in a solution containing sodium dodecyl sulfate, acetic acid, thiobarbituric acid, and water for 1 h at 95 °C. After cooling, water, n-butanol, and pyridine were added, and the mixture was centrifuged at 3000 rpm. The absorbance was measured at 532 nm [43].

### 2.10. Measurement of Nitrite Levels

Brain tissues were homogenized in phosphate buffer (pH 7.6). Griess reagent was added, and the mixture was incubated for 30 min. The absorbance was measured at 548 nm [43].

### 2.11. Enzyme-Linked Immunosorbent Assay

IL6, TNF-α, IL-1β, and Aβ levels were determined using an ELISA kit (Diaclone Research, Biosource Europe, USCN life Sciences) [45,46].

### 2.12. Histological Examination

For histopathological investigations, brain tissues were fixed at room temperature in a buffered formaldehyde solution (10% in PBS) [47,48]. Coronal sections of 5 μm thickness were obtained from the perilesional brain area of each animal and were evaluated by an experienced histopathologist. Histological sections were stained with H&E and evaluated using a Leica DM6 microscope (Leica Microsystems SpA, Milan, Italy) equipped with a motorized stage and associated with Leica LAS X Navigator software (Leica Microsystems SpA, Milan, Italy). Histopathologic scores of the damaged cortical area were evaluated as described previously: 0, no lesion observed; 1, gray matter contained one to five eosinophilic neurons; 2, gray matter contained five to 10 eosinophilic neurons; 3, gray matter contained more than 10 eosinophilic neurons; 4, small infarction (less than one-third of the gray matter area); 5, moderate infarction (one-third to one-half of the gray matter area); 6, large infarction (more than half of the gray matter area) [49]. In addition, neurons stained with H&E were also analyzed to reveal cell death in the hippocampal CA3 area. Cells presenting with nuclear and cytoplasmic staining were manually counted in the CA3 neurons. CA3 cell counting spanned the whole CA3 area, starting from the end of hilarneurons to the beginning of the curvature of the CA2 [15]. Neurons have euchromatin in the nucleus, a clearly visible nucleolus with surrounding cytoplasm. Neurons in the hippocampus have a characteristic pyramidal morphology. The scores from all the sections of each brain were averaged to obtain a final score for each mouse. All the histological studies were performed in a blinded fashion.

### 2.13. Western Blot Analysis

Western blots were performed on whole brain and hippocampus as already described [50,51]. Specific primary antibodies, i.e., anti-ikb-α (Santa Cruz Biotech, sc-1643), or anti-NFkB (Santa Cruz Biotechnology, sc-8008), or anti-Nrf2 (Santa Cruz Biotechnology, sc-36594), or anti-HO-1 (Santa Cruz Biotechnology, sc-136970), or anti-p-Tau (Santa Cruz Biotechnology, sc-32275). or anti-APP (Santa Cruz Biotechnology, sc-32277) were mixed in a 5% *w/v* nonfat dried milk solution and were incubated at 4 °C, overnight. Afterwards, blots were incubated with a peroxidase-conjugated bovine anti-mouse IgG secondary antibody or a peroxidase-conjugated goat antirabbit IgG (Jackson Immuno Research) for 1 h at room temperature [52,53]. To verify the amounts of protein were equal, membranes were also incubated with an antibody against β-actin (Santa Cruz Biotechnology). Signals were detected with an enhanced chemiluminescence detection system reagent (Super-Signal West Pico Chemiluminescent Substrate, Pierce) [54,55]. The relative expression of the protein bands was quantified by densitometry with Bio-Rad ChemiDoc XRS software and standardized to β-actin levels [56]. Images of blot signals were imported to an analysis software (Image Quant TL, v2003).

### 2.14. Statistical Evaluation

All values are expressed as mean ± standard error of the mean (SEM) of N observations. For in vivo studies, N represents the number of animals used. Results were analyzed by one-way ANOVA followed by a Bonferroni post-hoc test for multiple comparisons. A *p*-value of less than 0.05 was considered significant. * *p* < 0.05 vs. sham, # *p* < 0.05 vs. vehicle, ** *p* < 0.01 vs. sham, ## *p* < 0.01 vs. vehicle, *** *p* < 0.001 vs. sham, ### *p* < 0.001 vs. vehicle.

## 3. Results

### 3.1. Effect of Hidrox^®^—Histological Analysis after TBI

**Figure 1:**
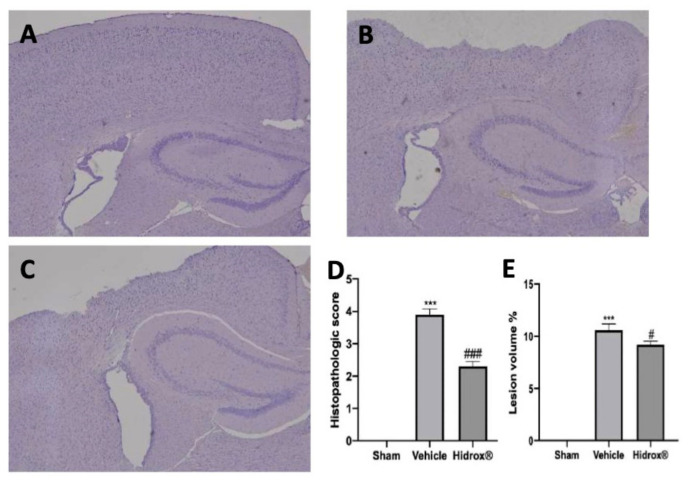
Hidrox^®^ administration reduced TBI-induced histological lesions. Histological analysis: Sham (**A**), Vehicle (**B**), Hidrox^®^ (**C**), Histopathological score (**D**), Lesion Volume % (**E**). For the analyses, *n* = 5 animals in each group were employed. (**C**) Results were analyzed by one-way ANOVA followed by a Bonferroni post-hoc test for multiple comparisons. Histopathological score (**D**): F(2,12) = 110.8, Lesion Volume % (**E**): F(2,12) = 139.6. A *p*-value of less than 0.05 was considered significant. # *p* < 0.05 vs. vehicle, *** *p* < 0.001 vs. sham, ### *p* < 0.001 vs. vehicle.

Histological analysis of the perilesional area showed significant edema and tissue damage in samples from vehicle-treated rats (Figure 1B,D) compared to the sham samples (Figure 1A,D). Hidrox^®^ treatment reduced tissue injury in the perilesional area (Figure 1C,D) and the lesion volume (Figure 1E).

### 3.2. Effect of Hidrox^®^ Treatment on Oxidative Hippocampal Alterations

**Figure 2:**
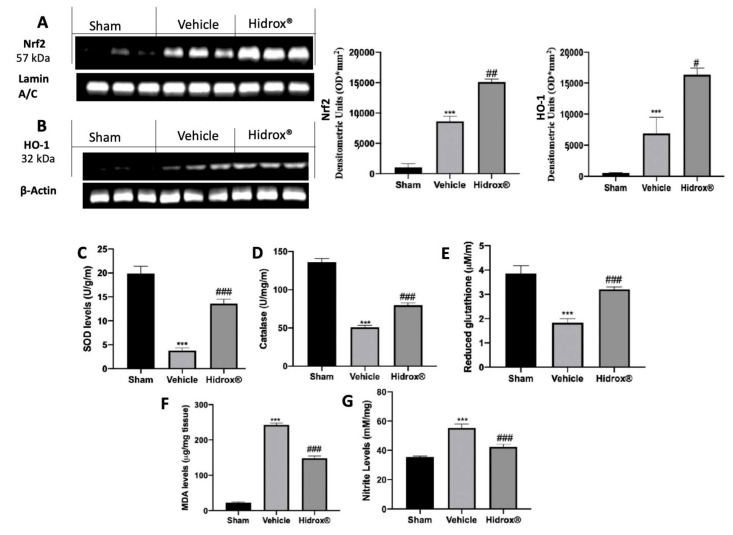
Hidrox^®^ administration reduced prooxidative alterations. Western blot analysis in whole brain of Nrf2 (**A**), HO-1 (**B**), SOD levels (**C**), Catalase levels (**D**), Reduced glutathione (**E**), Malondialdehyde (MDA) levels (**F**), Nitrite levels (**G**). For the analyses, *n* = 5 animals in each group were employed. Results were analyzed by one-way ANOVA followed by a Bonferroni post-hoc test for multiple comparisons. Densitometric analysis of a representative western blot analysis of Nrf2 expression (**A**): F (2,6) = 112.2, Densitometric analysis of a representative western blot analysis of HO-1 expression (**B**): F (2,6) = 24.07, SOD levels (**C**): F (2,12) = 40.68, Catalase levels (**D**): F(2,12) = 90.28, Reduced glutathione (**E**): F(2,12) = 27.57, Malondialdehyde (MDA) levels (**F**): F (2,12) = 177.8, Nitrite levels (**G**): F(2,12) = 50.31. A *p*-value of less than 0.05 was considered significant. # *p* < 0.05 vs. vehicle, ## *p* < 0.01 vs. vehicle, *** *p* < 0.001 vs. sham, ### *p* < 0.001 vs. vehicle.

In order to evaluate the antioxidant properties of Hidrox^®^, Western Blot analyses were conducted to assess the activation of the Nrf2 pathway. Samples from Hidrox^®^-treated animals showed increased Nrf2 nuclear expression compared to tissues harvested from vehicle-treated rats (Figure 2A). Well in line with this result, HO-1 expression as increased by Hidrox^®^ administration as compared to the vehicle-treated group. Additionally, the levels of SOD, catalase, and GSH were determined. Vehicle-treated rats showed a significant decrease in SOD, Catalase, and GSH compared to the sham animals. Treatment with Hidrox^®^ resulted in a significant reduction of SOD (Figure 2C), Catalase (Figure 2D), and GSH (Figure 2E) levels. Moreover, Hidrox^®^ administration reduced lipid peroxidation (Figure 2F) and nitrite levels (Figure 2G) compared to the vehicle-treated rats.

### 3.3. Effect of Hidrox^®^ Treatment on Cytokine Expression and NFkB Pathway

**Figure 3:**
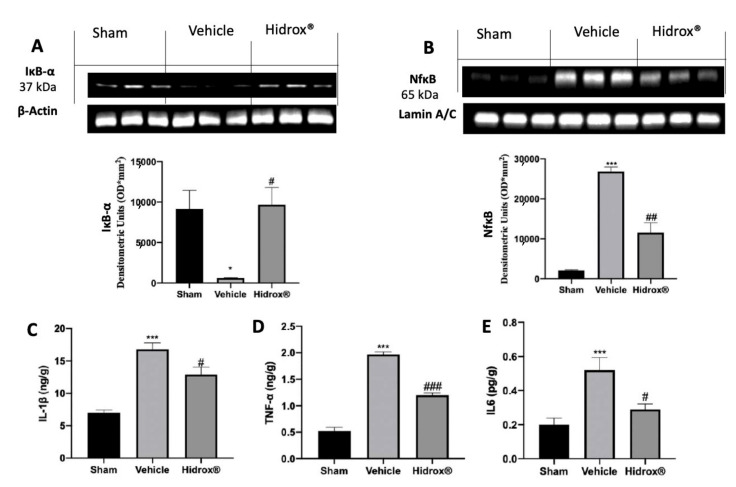
Hidrox^®^ administration reduced the levels of cytokines. Western blot analysis in whole brain of IkB-α (**A**) and NFkB (**B**) expressions, IL-1β levels (**C**), TNF-α levels (**D**), IL6 levels (**E**). For the analyses, *n* = 5 animals in each group were employed. Results were analyzed by one-way ANOVA followed by a Bonferroni post-hoc test for multiple comparisons. Densitometric analysis of a representative western blot analysis of IkB-α expression (**A**): F (2,6) = 7.896, Densitometric analysis of a representative western blot analysis of NFkB expression (**B**): F (2,6) = 63.78, IL-1β levels (**C**): F(2,12) = 14.83, TNF-α levels (**D**): F (2,12) = 92.69, IL6 levels (**E**): F(2,12) = 12.05. A *p*-value of less than 0.05 was considered significant. * *p* < 0.05 vs. sham, # *p* < 0.05 vs. vehicle, ## *p* < 0.01 vs. vehicle, *** *p* < 0.001 vs. sham, ### *p* < 0.001 vs. vehicle.

To test the anti-inflammatory properties of Hidrox^®^, Western Blot analyses were conducted on IkB-α and NFkB expression in the cytosol and nucleus, respectively. TBI in vehicle-treated rats decreased IkB-α expression in the cytosol compared to the expression in the sham rats. In line with this result, in the vehicle-treated group, the expression of NFkB was increased in the nuclear compartment compared to the sham group. Hidrox^®^ treatment significantly increased IkB-α levels in the cytosol (Figure 3A) and reduced NFkB expression in the nucleus (Figure 3B). Next, we estimated the anti-neuroinflammatory effect of Hidrox^®^ administration by evaluating cytokine expression. TBI induced an increase of IL-1β (Figure 3C), TNF-α (Figure 3D), and IL6 (Figure 3E) levels in the vehicle-treated group as compared to the sham rats. Hidrox^®^ treatment significantly reduced pro-inflammatory cytokines expression.

### 3.4. Effect of Hidrox^®^ Treatment on Behavioral Alterations

**Figure 4:**
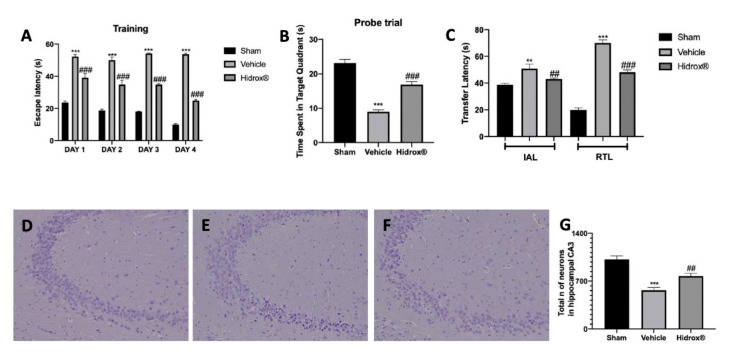
Hidrox^®^ administration reduced TBI-induced behavioral alterations. Morris Water Maze Test: Training (**A**), Probe Trial (**B**); Elevated Plus Maze Test (**C**), Histological analysis of the hippocampal CA3 area: Sham (**D**), Vehicle (**E**), Hidrox^®^ (**F**), Total number of neurons (**G**). For the analyses, *n* = 5 animals in each group were employed. Results were analyzed by two-way ANOVA followed by a Bonferroni post-hoc test for multiple comparisons. Morris Water Maze Test: Training (**A**): F (6,48) = 8.675. The results were analyzed by one-way ANOVA followed by a Bonferroni post-hoc test for multiple comparisons. Probe Trial (**B**): F (2,12) = 71.14; Elevated Plus Maze Test (**C**): F(5,24) = 57.90, Total number of neurons (**G**): F(2,12) = 22.10. A *p*-value of less than 0.05 was considered significant. ** *p* < 0.01 vs. sham, ## *p* < 0.01 vs. vehicle, *** *p* < 0.001 vs. sham, ### *p* < 0.001 vs. vehicle.

MWM was employed for evaluating spatial learning. In the training period (Figure 4A), all groups showed a decreasing trend in the escape latency on day 4 as compared to day 1. In the probe trial, Hidrox^®^ treatment significantly increased the time spent in the target quadrant, indicating the degree of memory consolidation after learning, which was decreased by TBI (Figure 4B). In the elevated plus maze test, we evaluated memory-related processes. In both IAL and RTL, vehicle-treated rats showed increased transfer latency compared to the sham animals. Hidrox^®^ administration reduced the time of transfer latency in IAL and RTL (Figure 4C). An increase in RTL demonstrates that TBI induced a marked impairment in learning and memory. In contrast, treatment with Hidrox^®^ led to a significant decrease in transfer latency as compared to vehicle-treated group, indicating an improvement in the retention of memory.

During the behavioral analysis, we checked the confounding factors that would interfere with the results of the analysis. In regard to motor impairment, we did not find any difference in motor function between the three groups and, in particular, between the sham group and the animals subjected to TBI. Our results are well in line with the literature [55]. In regard to anxiety, measured in the elevated plus maze test, the time spent in the open and closed arms recorded for 5 min is usually employed to evaluate anxiety-like behavior [56]. Our elevated plus maze test was performed for a maximum time of 90 sec. No significant difference between the three groups and, in particular, between the sham group and the animals subjected to TBI, was detected in the time spent in the two arms.

Histological analysis of the hippocampal CA3 area showed a reduced number of neurons in samples from vehicle-treated rats (Figure 4E,G) compared to samples from the sham group (Figure 4D,G). The animals treated with Hidrox^®^ showed an increased number of neurons in the hippocampal CA3 area (Figure 4F,G).

### 3.5. Effect of the Hidrox^®^ Treatment on AD-Like Neuropathology

**Figure 5:**
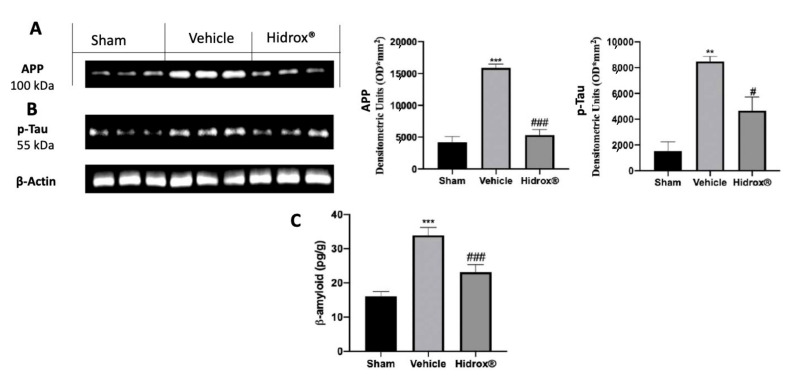
Hidrox^®^ administration reduced AD markers. Western blot analysis of hippocampus p-Tau (**A**) and APP (**B**) expression and β-amyloid levels (**C**). For the analyses, *n* = 5 animals in each group were employed. The results were analyzed by one-way ANOVA followed by a Bonferroni post-hoc test for multiple comparisons. Densitometric analysis of representative western blot analysis of APP expression (**A**): F (2,6) = 62.56, Densitometric analysis of representative western blot analysis of p-Tau expression (**B**): F (2,6) = 20.40, β-amyloid levels (**C**): F (2,12) = 16.43. A *p*-value of less than 0.05 was considered significant. # *p* < 0.05 vs. vehicle, ** *p* < 0.01 vs. sham, *** *p* < 0.001 vs. sham, ### *p* < 0.001 vs. vehicle.

Western blot analysis showed increased hippocampal p-Tau (Figure 5A) and APP (Figure 5B) expression in tissues harvested from vehicle-treated rats compared to those from the sham group. Hidrox^®^ significantly reduced the expression of both markers in the hippocampus (Figure 5A,B). Vehicle-treated rats showed increased β-amyloid accumulation compared to the sham animals. The Hidrox^®^ treatment significantly reduced β-amyloid accumulation (Figure 5C).

## 4. Discussion

Our study demonstrated the protective effects of Hidrox^®^ administration on the propagation of secondary damage in brain areas proximal and remote to the primary region of the injury in the late phase of TBI, characterized by AD-like features. Among the issues leading to TBI outcomes, the biochemical mechanisms causing oxidative stress are the well studied [3]. The increased ROS production following TBI has been shown to cause oxidative damage to cellular proteins, lipids, and cell membrane polyunsaturated fatty acids [18]. In particular, the role of Nrf2 in the defense against TBI-induced neuroinflammation is of particular interest [57,58,59,60]. Many pieces of evidence confirm the role of the Nrf2 pathway in modulating both oxidative stress and inflammation [61,62,63,64,65,66,67]. Hidrox^®^ is known to influence the promotion of the transcription of genes downstream of Nrf2 activation [41]. Nrf2 is an important genomic homeostatic regulator and a pleiotropic transcription factor that coordinates detoxification and anti-oxidative processes [19,68]. Hidrox^®^ administration upregulated the Nrf2 transcriptional system, inducing the activation of phase II detoxifying enzymes [19], such as HO-1, SOD, Catalase, and GSH, thus counteracting the neurodegenerative damage.

Well in line with the literature, the Hidrox^®^ treatment reduced lipid peroxidation and nitrite levels increased by TBI [60].

Oxidative stress damage aggravates neuronal injury promoting the inflammatory reaction and stimulating cytokine overexpression [69,70]. In particular, NFkB binding sites have been identified in the promoter region of the Nrf2 gene, which suggests a cross-talk between these two mechanisms in the inflammatory process [69]. In this paper, we showed Hidrox^®^ ability to increase IkB-α cytosolic expression and, in turn, to reduce NFkB nuclear expression [71,72,73,74,75,76]. Hidrox^®^ administration significantly reduced IL-1β, TNF-α, and IL6 levels, which are significantly enhanced following brain damage.

Finally, laboratory results from acute and chronic studies displayed that TBI can lead to progressive pathological alterations reflected in both behavior and brain structure, characterized by tissue atrophy, progressive hippocampal cell loss, and compromised neurotransmission combined with cognitive decline, which increase in the chronic stage of injury [77]. TBI has been associated with chronic neurodegenerative disorders. Several cognitive deficits have been described in TBI patients with AD-like symptoms [78,79,80,81].

The literature reports that this neurodegenerative process involves aberrant APP and phosphorylated-Tau overexpression accompanied by β-amyloid accumulation and persistent neuron loss in the hippocampal CA3 area [10,15]. Moreover, increased APP expression, in tandem with reduced neurogenesis in the hippocampus, results in impaired hippocampal-mediated cognitive activity [82,83]. Notably, robust hippocampal plasticity is thought to play a role in learning and memory consolidation, which is an important aspect of cognitive function [84,85,86]. This hippocampal neurodegeneration, which is responsible for many types of cognitive impairment typical of AD, is equally present in TBI and may support the evolution of memory and learning deficits in TBI. In particular, various studies indicate that progressive amyloidosis and APP overexpression, neurons loss in the hippocampal CA3 area, and related cognitive impairment are not transitory, but a permanent sequela of TBI [87,88,89,90,91].

Our data, well in line with the literature, showed a significantly decreased number of hippocampal neurons in the CA3 region post-TBI, while Hidrox^®^ treatment was associated with a significantly reduced damage in the CA3 area. In particular, Hidrox^®^ decreased AD-like phenotypic markers such as β-amyloid accumulation and APP and p-Tau overexpression. From a behavioral point of view, Hidrox^®^ treatment ameliorated TBI-induced cognitive dysfunction and memory impairment.

## 5. Conclusions

This study suggests that treatment with the antioxidant and anti-inflammatory Hidrox^®^ could be promising to reduce inflammation and AD-like diseases resulting from neuroinflammatory responses induced by primary and secondary injuries.

## Data Availability

The data presented in this study are available on request from the corresponding author.
